# 

**Published:** 1859-08

**Authors:** 


### CONTENTS.

### ORIGINAL COMMUNICATIONS.

PAGE.

ARTICLE I.—Professional Ollapodrida. By A RETIRED PHARMACIEN,........ 709

ARTICLE II.—An Essay on Child-Bed Fever, read before the Medical Association of the State of Georgia. By S. H. DEAN, M. D., Conyers Ga.,.. 719

ARTICLE III.—Reports of Cases, occurring in "Bellevue Hospital," Richmond, Va. By THOMAS POLLARD, M. D., one of the Physicians to the Hospital, ........ 725

ARTICLE IV.—" Bibron's Antidote." By D. O. C. HEERY, M. D., Atlanta, Georgia,........ 737

ARTICLE V.—Prevention of Pitting in Small-Pox. By T. S. DENNY, M. D., of Atlanta, Georgia,........ 733

ARTICLE VI.—Cinchonia in Menorrhagia—Case. By ROBERT BATTEY, M. D., of Rome, Georgia,........ 734

ARTICLE VII.—Strangulated Hernia Reduced by a Cold Douche—Translation. By F. H. EVANS, M. D., Aberdeen, Mississippi,........ 736

ARTICLE VIII.—Croupal Diphtheria. By F. H. EVANS, M. D., Aberdeen, Mississippi,........ 738

ARTICLE IX.—Dentition. By J. P. H. BROWN, Dentist, Atlanta, Ga.,........ 740

### SELECTIONS.

Obesrvations on Diphtheria. By STEPHEN MONCKTON, M, D.,........ 744

Monthly Review of Parisian Medicine and Surgery. By THEOPHILUS MACK, M. D., Lecturer on Materia Medica and Therapeutics in the Medical Department of the University of Buffalo,........ 757

### EDITORIAL AND MISCELLANEOUS.

An Original Number,........ 763

New York Correspondence,........ 763

Southern Field and Fireside,........ 764

Bibliographical, ........ 765

Inverson of the Womb. By WALTER CHANNING, M. D.,........ 769

List of Payments, ........ 771

University of Louisville,........ 771

Metereological Table,........ 772

